# Three-Dimensional Spinal and Pelvic Alignment as Determinants of Anticipatory Core Muscle Activation

**DOI:** 10.3390/jcm14238432

**Published:** 2025-11-27

**Authors:** Maryam M. Abdellatif, Ibrahim M. Moustafa, Abdulrahman M. Alsubiheen, Mishal M. Aldaihan, Iman Akef Khowailed

**Affiliations:** 1 Department of Physiotherapy, College of Health Sciences, University of Sharjah, Sharjah 27272, United Arab Emirates; mabdellatif@sharjah.ac.ae (M.M.A.); iabuamr@sharjah.ac.ae (I.M.M.); 2 Neuromusculoskeletal Rehabilitation Research Group, RIMHS—Research Institute of Medical and Health Sciences, University of Sharjah, Sharjah 27272, United Arab Emirates; 3 Faculty of Physical Therapy, Cairo University, Giza 12613, Egypt; 4 Department of Rehabilitation Health Sciences, College of Applied Medical Sciences, King Saud University, P.O. Box 10219, Riyadh 11433, Saudi Arabia; mishaldaihan@ksu.edu.sa

**Keywords:** 3D spinal alignment, vertebral rotation, anticipatory postural adjustments, feedforward activation, core stability

## Abstract

**Background/Objectives**: Three-dimensional (3D) spinal and pelvic alignment plays a critical role in maintaining anticipatory postural control. However, the extent to which specific multiplanar alignment parameters influence feedforward activation of trunk stabilizing muscles remains unclear. This study aimed to determine whether sagittal, coronal, and transverse postural deviations predict anticipatory muscle activation patterns during externally induced perturbations. **Methods**: Surface electromyography (EMG) was recorded from bilateral external oblique (EO), lumbar multifidus (LM), and transversus abdominis/internal oblique (TrA/IO) muscles in 100 asymptomatic young adults (18–25 years) performing dynamic right-leg raises. Spinal and pelvic alignment was quantified using rasterstereography, including sagittal and coronal imbalance, pelvic tilt, torsion, rotation, vertebral rotation, and spinal curvatures (kyphotic and lordotic angles). Regression models examined how these parameters predicted EMG onset latency and activation amplitude. **Results**: Distinct alignment patterns were associated with altered anticipatory control. Increased vertebral rotation and greater sagittal imbalance were linked to delayed activation of EO and LM, while asymmetries in pelvic torsion and tilt were related to less efficient TrA/IO recruitment. Conversely, more balanced spinal curvatures corresponded with earlier, more coordinated muscle activation across the trunk. **Conclusions**: Multiplanar spinal and pelvic alignment significantly influences anticipatory neuromuscular strategies. Identifying how specific postural deviations disrupt feedforward activation provides a functional basis for targeted rehabilitation programs aiming to restore alignment, enhance trunk stability, and prevent recurrent postural dysfunction.

## 1. Introduction

Feedforward activation, also known as anticipatory postural adjustment (APA), is a fundamental neuromuscular mechanism that underpins postural stability and coordinated movement [1,2]. It involves the pre-activation of deep stabilizing musculature prior to voluntary limb motion, allowing the body to counteract destabilizing forces and preserve spinal integrity [1,2]. Electromyographic (EMG) studies demonstrate that trunk stabilizers such as the transversus abdominis (TrA) and multifidus activate approximately 30–50 ms before prime movers like the deltoid, highlighting the predictive control of the central nervous system (CNS) over posture and movement [3,4]. This anticipatory strategy ensures proximal stability during distal motion and contributes to mechanical efficiency, reduced injury risk, and enhanced movement precision [5].

Proprioceptive and somatosensory inputs play a pivotal role in refining APA timing and magnitude. Afferent feedback from muscles, joints, and ligaments informs internal models of motor planning and facilitates adaptive calibration of anticipatory responses [6,7]. When proprioceptive signaling is disrupted because of chronic pain, altered loading, or structural misalignment, feedforward activation may be delayed or diminished, resulting in compensatory overactivation of superficial musculature and increased mechanical strain on the spine [8,9]. Individuals with spinal dysfunction often exhibit delayed recruitment of deep trunk muscles and reduced anticipatory control, both of which compromise spinal stability and elevate the risk of further musculoskeletal impairment [7,10].

While the neurophysiological mechanisms of APAs are well established, the influence of three-dimensional (3D) spinal and pelvic alignment on anticipatory neuromuscular control remains insufficiently explored. Previous research has predominantly examined isolated or two-dimensional postural parameters, leaving the integrated, multiplanar contributions of vertebral rotation, sagittal imbalance, and pelvic asymmetries to feedforward muscle activation inadequately defined. Structural deviations such as sagittal or coronal imbalance, vertebral rotation, pelvic torsion, and alterations in kyphotic or lordotic curvature are not merely anatomical anomalies; they alter segmental alignment, redistribute mechanical loads across vertebrae and pelvic joints, and modify proprioceptive input from muscle spindles, joint capsules, and ligaments [11,12,13]. These changes can in turn affect neuromuscular recruitment strategies, including the timing, magnitude, and coordination of trunk muscle activation, as the CNS adjusts feedforward and feedback control to maintain postural stability during voluntary or externally triggered movements [3,9,14].

From a biomechanical standpoint, 3D spinal and pelvic alignment defines the geometric and mechanical relationships between the spine and pelvis, directly influencing the functional efficiency of anticipatory control. Altered alignment modifies moment arms, changes muscle length–tension relationships, and affects load transfer across spinal segments. These deviations may disturb proprioceptive accuracy and timing of core muscle activation, leading to reduced mechanical efficiency, compensatory recruitment of superficial muscles, and suboptimal stabilization strategies. Consequently, understanding these biomechanical determinants provides crucial insight into how structural variability can impair or enhance feedforward postural mechanisms.

Clinically, such biomechanical alterations carry significant implications for both injury prevention and rehabilitation. Impaired anticipatory control secondary to postural deviation can predispose individuals to repetitive micro-strain, inefficient stabilization, and chronic pain syndromes. Conversely, restoring optimal alignment has been shown to normalize neuromuscular activation patterns and improve trunk coordination [15,16,17]. Systematic reviews also demonstrate that altered lumbopelvic kinematics are associated with impaired movement coordination and increased compensatory muscle overuse [18]. Therefore, delineating how specific alignment parameters affect feedforward control can inform the design of targeted rehabilitation strategies aimed at correcting posture, enhancing proprioceptive feedback, and optimizing neuromuscular efficiency in both preventive and therapeutic contexts [19].

Given these insights, investigating how multiplanar spinal and pelvic alignment parameters predict feedforward activation characteristics is of substantial biomechanical and clinical relevance. Determining the predictive value of specific alignment factors—such as vertebral rotation, sagittal imbalance, and pelvic asymmetries—for onset latency and activation amplitude could clarify the structural determinants of anticipatory postural control. Accordingly, this study aimed to examine the predictive role of multiplanar spinal and pelvic alignment variables in shaping feedforward activation of key core stabilizing muscles during dynamic perturbation tasks in healthy young adults. It was hypothesized that greater deviations in spinal and pelvic alignment—particularly increased vertebral rotation and sagittal imbalance—would be associated with delayed activation onset and reduced amplitude of deep trunk stabilizers.

By addressing this relationship, the study provides an applied framework for understanding the structural–neuromuscular interface underlying trunk stabilization. Its findings may contribute to more precise assessment of postural alignment and guide evidence-based interventions that enhance anticipatory postural control and reduce the risk of future dysfunction.

## 2. Materials and Methods

### 2.1. Study Design and Overview

A cross-sectional predictive design was employed to examine the relationship between 3D spinopelvic alignment parameters and the anticipatory activation characteristics of core stabilizing muscles in healthy young adults. The study was conducted at the Biomechanics and Rehabilitation Research Laboratory, University of Sharjah, following approval by the institutional Research Ethics Committee (REC-25-01-23-01-PG), approval date: 15 May 2025. All participants provided written informed consent before participation. Data were collected between 19 May and 28 August 2025, under controlled laboratory conditions.

### 2.2. Variable Classification and Conceptual Hierarchy

To enhance conceptual transparency and methodological clarity, the study variables were organized into three hierarchical categories: predictor variables, dependent variables, and covariates/control factors.

#### 2.2.1. Predictor Variables (Independent)

Predictor variables consisted of the 3D spinopelvic alignment parameters derived from DIERS Formetric 4D^®^ (v3.17.0.27) rasterstereography. The measured parameters included:

- Sagittal imbalance (mm)
- Coronal imbalance (mm)
- Kyphotic angle (°)
- Lordotic angle (°)
- Vertebral rotation (°)
- Pelvic rotation (°)
- Pelvic torsion (°)
- Pelvic drop (tilt) (°)

These variables were selected for their biomechanical relevance to trunk posture and potential influence on anticipatory neuromuscular activation.

#### 2.2.2. Dependent Variables (Outcomes)

The dependent variables represented the electrophysiological measures of anticipatory postural control obtained from surface EMG.

Two parameters were analyzed for each recorded trunk muscle—transversus abdominis/internal oblique (TrA/IO), lumbar multifidus (LM), and external oblique (EO):

1. Onset latency (ms): The time difference between the onset of trunk-muscle activation and that of the prime mover (rectus femoris [RF]), with negative values indicating anticipatory activation.
2. Activation amplitude (%MVIC): The root-mean-square (RMS) EMG amplitude normalized to each participant’s maximal voluntary isometric contraction (MVIC).

These indices quantify the timing and magnitude of feedforward control and serve as established markers of anticipatory stabilization efficiency.

#### 2.2.3. Covariates and Control Factors

Several covariates were addressed both procedurally and statistically to reduce potential confounding influences:

- Age and physical condition: Participants were restricted to healthy young adults aged 18–25 years with normal body mass index (BMI) (mean ± SD = 23.5 ± 2.6 kg/m^2^). This narrow demographic window reduced interindividual variability related to neuromuscular performance and habitual activity.
- Sex: Equal numbers of male and female participants (*n* = 50 each) were included. Preliminary tests indicated no significant sex differences in spinopelvic or EMG variables (*p* > 0.05), except for a marginal latency difference in the right TrA/IO (*p* = 0.044). Accordingly, sex was not included as a fixed factor but was verified as a non-significant source of variance through sensitivity testing.
- Limb dominance: Only right-leg-dominant participants were included to standardize lateralized recruitment patterns.
- BMI and body composition: BMI was recorded and extreme values (>27 kg/m^2^) were excluded to minimize EMG attenuation due to subcutaneous fat thickness.
- Fatigue and physical activity control: Participants were instructed to refrain from vigorous activity for 24 h before data collection. Each trial was separated by a 30 s rest interval to eliminate the potential effects of localized fatigue.

Together, these procedural and statistical controls minimized demographic, anthropometric, and physiological variability, ensuring that the relationships identified between alignment and muscle activation reflected true biomechanical associations.

### 2.3. Participants

A total of 100 healthy young adults (50 males and 50 females; aged 18–25 years), were recruited through convenience sampling from the student and staff population of the university. Participants were included if they were between 18 and 25 years of age, had no current or previous history of neck, back, neurological, or orthopedic disorders, had not undergone any spinal or lower-limb surgery, and demonstrated right-leg dominance, which was determined by asking which leg they would use to kick a ball. The inclusion of only right-leg dominant individuals was intended to standardize limb dominance, reduce between-subject variability, and minimize potential confounding effects of mixed dominance on EMG onset and amplitude measures. Exclusion criteria included the presence of neurological disorders, musculoskeletal deformities, systemic diseases affecting mobility, current pain or musculoskeletal injuries, or pregnancy.

The required sample size was estimated a priori using Cohen’s formula for multiple linear regression, with eight predictor variables, a moderate effect size (*f*^2^ = 0.15), a two-tailed alpha of 0.05, and 80% power. The calculation indicated a minimum of 62 participants. To improve model stability and account for potential data loss, the sample was increased to 100 participants, exceeding the recommended minimum of 10–15 observations per predictor. Post hoc power analysis using G*Power (v3.1) confirmed that this sample size provides >0.90 power to detect moderate effects, supporting its adequacy for the planned analyses.

### 2.4. Spinopelvic Alignment Assessment

Static postural parameters were measured using the DIERS Formetric 4D^®^ rasterstereography system (DIERS International GmbH, Schlangenbad, Germany), a validated, non-invasive optical surface topography device that captures high-resolution surface data of the back without radiation exposure and reconstructs a 3D model of the spine and pelvis based on optical triangulation. The system is highly reliable and reproducible for 3D spinal and pelvic alignment measurement, with previous validation studies reporting intra-rater intraclass correlation coefficient (ICC) values between 0.91 and 0.98, inter-rater ICC values between 0.89 and 0.96, and a standard error of measurement (SEM) approximately 0.6–3.1 mm for trunk inclination and 0.65–1.2° for curvature angles [20,21]. Calibration was automatically performed before each scanning session using the manufacturer’s three-point reference protocol, ensuring correct alignment of the optical triangulation axes. Each participant completed a familiarization session that included 2–3 practice scans to ensure postural stability before data recording commenced. The following postural parameters were extracted as continuous predictor variables for subsequent analysis: sagittal imbalance (anterior–posterior trunk shift relative to the pelvis), coronal imbalance (lateral deviation in the frontal plane), pelvic rotation (transverse plane rotation of the pelvis), pelvic torsion (asymmetrical rotation between the right and left hemipelvis), pelvic obliquity (vertical displacement difference between the iliac crests), vertebral rotation (axial rotation of the spinal column), thoracic kyphosis angle, lumbar lordosis angle, and pelvic tilt. These parameters were selected based on their biomechanical relevance to spinopelvic alignment and potential influence on neuromuscular control.

**Figure 1:**
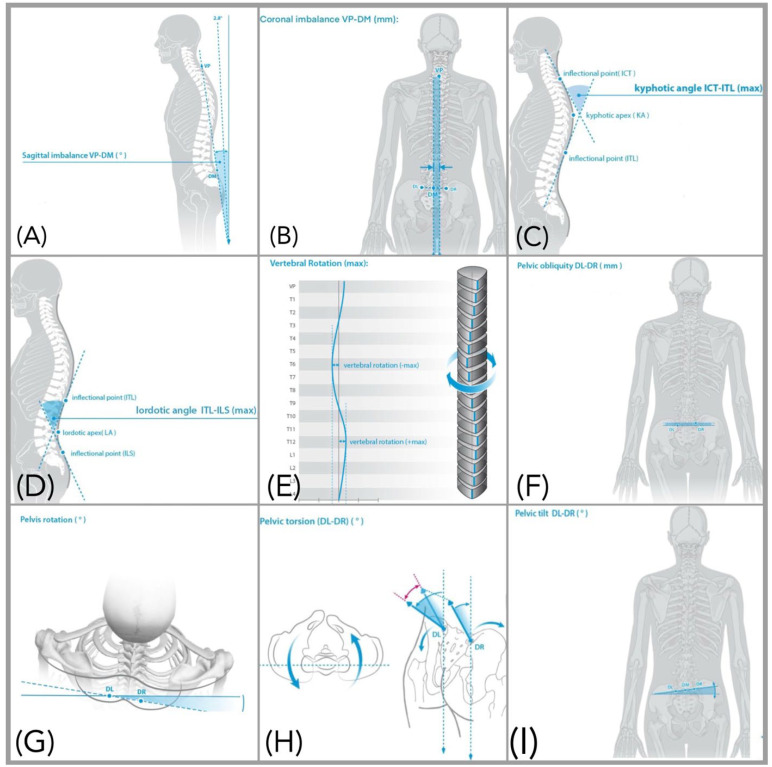
Normalized Rasterstereographic visualization of spino-pelvic alignment parameters: (**A**) Sagittal imbalance, (**B**) Coronal imbalance, (**C**) Kyphotic angle, (**D**) Lordotic angle, (**E**) Vertebral rotation, (**F**) Pelvic obliquity, (**G**) Pelvic rotation, (**H**) Pelvic torsion, (**I**) Pelvic tilt. With permission from DIERS Formetric 4D^®^ (DIERS International GmbH, Schlangenbad, Germany).

All participants were instructed to refrain from strenuous physical activity for 24 h prior to testing to minimize fatigue-related alterations in posture. Upon arrival, participants removed their upper body clothing and footwear and wore shorts; female participants were provided with a gown secured anteriorly with medical tape to prevent obstruction of posterior landmarks. All jewelry, accessories, and long hair were removed or secured to avoid interference with data acquisition. For accurate assessment, reflective markers were carefully placed over the spinous process of C7 and bilaterally on the posterior superior iliac spine. Marker placement accuracy was verified with an initial static scan of the back surface. Once confirmed, the markers remained in place throughout the static assessment, and rasterstereographic scanning commenced. Spinal and pelvic alignment parameters are defined in Table 1, with their sample means and standard deviations, and visually represented in Figure 1.

### 2.5. Electromyographic Measurement

Surface EMG was recorded using the NeXus Q32 system (MindMedia NeuroLOGX B, Herten, Netherlands). Electrode placement was verified through palpation and real-time monitoring. Pilot testing on 10 participants confirmed high reliability, with intra-session ICC values of 0.89–0.95 for normalized %MVIC amplitudes and <5 ms onset detection variance [22,23].

#### 2.5.1. Experimental Task and Setup

Participants stood barefoot on a flat surface, with feet shoulder-width apart, arms relaxed at their sides, and eyes focused on a visual target placed at eye level. Participants performed 10 trials of an externally cued, rapid single right leg lifts:

- Upon hearing an auditory cue, participants flexed their right hip to 90° as quickly as possible.
- Upon a second auditory cue, participants gradually returned to the starting position.

Participants were instructed to avoid any compensatory trunk movements during the task. Each trial was separated by a 30 s rest interval to minimize fatigue. The time intervals between auditory cues were varied to reduce anticipation. To ensure consistency and minimize learning effects, participants performed up to 10 practice trials before the actual data collection.

During the externally cued, self-executed perturbation task, surface EMG was used to record muscle activity from the dominant and non-dominant TrA/IO, LM, and EO muscles, all of which play essential roles in feedforward postural control. These muscles were specifically selected because they are recognized as key contributors to APAs. Their early activation has been shown to stabilize the spine during voluntary limb movements, supporting trunk control through feedforward mechanisms [14].

#### 2.5.2. Electrode Placement

Surface EMG electrodes were placed following the Surface Electromyography for the Non-Invasive Assessment of Muscles (SENIAM) guidelines and validated anatomical references:

1. TrA/IO:
  o Placement: 2 cm medial to the anterior superior iliac spine (ASIS).
2. LM:
  o Placement: 2 cm lateral to the midline at the level of the L4 spinous process.
3. EO:
  o Placement: halfway between the iliac crest and the costal margin along the mid-axillary line at a slightly oblique angle.

Electrode placements for the LM muscle followed the SENIAM guidelines, while the TrA/IO and EO placements adhered to protocols outlined by [24]. Electrodes were aligned parallel to the orientation of the underlying muscle fibers to optimize signal quality and minimize noise. The grounding electrode was placed over the right iliac crest. Prior to electrode application, the skin was shaved, lightly abraded, and cleaned with alcohol to ensure impedance below 10 kΩ.

#### 2.5.3. EMG Signal Acquisition and Processing

Surface EMG signals were recorded at a sampling rate of 2048 Hz with a hardware band-pass filter (20–450 Hz) to suppress movement artifacts and electrical noise while preserving physiologically relevant signal components. Bipolar Ag/AgCl electrodes with a 20 mm inter-electrode distance were used to minimize crosstalk.

#### 2.5.4. Cross-Talk Mitigation and Signal Verification

To minimize potential cross-talk between the hip flexor compartment and the abdominal wall, TrA/IO electrode placement was verified through palpation during gentle abdominal hollowing to confirm localized activation without hip-flexor spillover. Leads were secured and routed caudally to minimize cable motion, with the ground electrode positioned over the right iliac crest to stabilize common-mode rejection. Participants practiced isolated TrA/IO hollowing and brief resisted hip flexion while EMG channels were monitored in real time to ensure that RF activity did not induce synchronous bursts on TrA/IO beyond baseline noise.

#### 2.5.5. Signal Preprocessing

Following acquisition, EMG signals were digitally processed offline in MATLAB (R2024a, MathWorks, Natick, MA, USA). Data were full-wave rectified and smoothed using a fourth-order low-pass Butterworth filter (cutoff ≈ 100 Hz) to reduce high-frequency noise while preserving onset characteristics. A 50 ms moving RMS window was then applied to stabilize the baseline for onset detection. Pilot testing confirmed that this window introduced <5 ms bias relative to a 20 ms window, ensuring temporal precision without compromising signal stability.

#### 2.5.6. Movement Onset Definition

Leg-raise initiation (T_0_) was operationalized a priori as the onset of RF EMG activity on the raised right limb. RF onset was detected from the full-wave rectified, 50 ms RMS smoothed RF signal using a baseline-referenced threshold: the first sample exceeding mean + 3 SD of a −400 to −200 ms pre-cue baseline and persisting for ≥25 ms. A 150 ms refractory window was enforced to prevent multiple detections within a trial. Two raters, blinded to group allocation, reviewed algorithmic detections; disagreements were resolved by consensus. All trunk-muscle latencies reported in this study are expressed relative to this RF onset (negative values = anticipatory activation).

#### 2.5.7. Onset Detection

Trunk-muscle onsets (EO, LM, TrA/IO) were detected using the integrated profile (IP) method adapted for trunk EMG [22]. For each muscle, the rectified EMG was cumulatively integrated over a detection window of −150 to +200 ms relative to RF onset; onset was the time of maximum vertical deviation between the IP curve and a straight reference line joining window endpoints.

Onset latency was calculated as:

$$Latency\left(ms\right)=Trunk\;Muscle\;Onset\;Time-RF\;Onset\;Time$$

Negative values indicate anticipatory (feedforward) activation preceding RF onset. Latencies were computed for each trial and then averaged across ten perturbation trials per muscle per participant.

#### 2.5.8. Surface EMG Amplitude Normalization

In this study, surface EMG signals were normalized to enable comparison of muscle activation amplitudes both within and between individuals during the feedforward activation task. The normalization process was based on the MVIC of each muscle, following internationally recognized protocols and adapted to the muscles tested (TrA/IO, LM and EO). The MVIC for each muscle was determined using a standardized position for manual muscle testing, ensuring uniformity in resistance and joint angles.

- TrA/IO: Participants performed an abdominal hollowing maneuver in crook lying, instructed to draw in the abdominal wall without pelvic or rib movement.
- LM: Participants lay prone and performed a contralateral arm and leg lift, while maintaining neutral lumbar lordosis.
- EO: Participants performed an isometric side-lying lateral trunk flexion against resistance, with resistance applied just above the iliac crest.

Each contraction lasted for 5 s and was performed three times, with a rest period of 30 to 60 s between trials to minimize fatigue. The peak RMS value recorded during any of the three trials for each muscle was chosen as the individual’s 100%MVIC reference for that specific muscle.

Considering the known challenges in achieving true maximal activation of deep trunk stabilizers during isolated MVIC tasks, the following factors were considered:

- Consistent verbal encouragement was provided throughout all MVIC efforts.
- The abdominal hollowing technique was emphasized for the TrA/IO, as abdominal bracing typically activates more superficial muscles, such as the rectus abdominis.
- Pilot tests were conducted on a selected group of participants to verify the reliability of MVIC measurements prior to the main data collection.

According to previous research [23], using MVIC for surface EMG normalization in core muscles demonstrates high intra-individual reliability and helps reduce inter-individual variability. Careful attention was given to consistent electrode placement, standardized instructions for contractions, and designated rest periods to enhance reproducibility across sessions and reduce measurement errors.

#### 2.5.9. Amplitude Analysis

Muscle activation amplitude was computed from the RMS-smoothed signal over a peri-onset window from −50 ms to +100 ms relative to RF onset, capturing anticipatory and early feedback activity. These amplitudes were normalized to the peak RMS value obtained during each muscle’s MVIC test and expressed as %MVIC, calculated as:

$$Normalized\;EMG\left(\%MVIC\right)=\frac{Mean\;RMS\;during\;task\;window}{Peak\;RMS\;during\;MVIC}\times 100$$

Mean %MVIC values per muscle per participant were calculated across ten trials to enhance reliability and reduce within-subject variability.

#### 2.5.10. Pipeline Consistency

All EMG preprocessing, onset detection, and amplitude calculations were performed in MATLAB (R2024a, MathWorks, Natick, MA, USA) using a single standardized pipeline for all participants. This workflow—combining rigorous electrode preparation, high-fidelity acquisition, defined filter parameters, validated onset detection, and standardized normalization—ensures maximal reproducibility and aligns with best practices for high-impact EMG and biomechanics research.

### 2.6. Outcome Measures

Primary outcomes were trunk muscle onset latencies relative to RF onset (ms) for EO, LM, and TrA/IO bilaterally. Negative latency values indicate anticipatory (feedforward) activation preceding RF onset, whereas positive values reflect delayed (feedback) activation occurring after prime mover onset. Additionally, EMG amplitude was analyzed to quantify the intensity of muscle activation, based on the peak amplitude within a defined time window, providing insight into recruitment patterns and compensatory strategies.

### 2.7. Statistical Analysis

All statistical analyses were conducted using SPSS (version 27.0; IBM Corp., Armonk, NY, USA) and MATLAB (R2024a; MathWorks, Natick, MA, USA). Reliability of both DIERS and EMG measurements was verified through pilot testing and supported by published ICC and SEM values. Calibration and task familiarization were performed before data collection. Statistical assumptions of linearity, normality, and independence were confirmed through diagnostic tests (Shapiro–Wilk, Levene’s, and residual analyses). Interaction terms and model fit indices (adjusted R^2^, F statistic, Akaike Information Criterion [AIC], and Bayesian Information Criterion [BIC]) were reported, and false discovery rate (FDR) correction was applied to control for multiple testing. Predictor, dependent, and control variables were thus clearly defined and verified, ensuring a transparent analytical framework and robust interpretability of the predictive models. Prior to modeling, data were screened for completeness, outliers, and adherence to parametric assumptions. The dataset was complete, with no missing observations or influential outliers. The Shapiro–Wilk test confirmed that residuals approximated a normal distribution (all *p* > 0.05) and Levene’s test indicated homogeneity of variances across factor levels.

#### 2.7.1. Regression Modeling

To investigate the predictive influence of multiplanar spinal and pelvic alignment on anticipatory muscle activation, a series of multiple linear regression analyses were performed separately for each EMG outcome variable, including activation amplitude (%MVIC) and onset latency (ms). The following postural predictors were entered simultaneously into each model: sagittal imbalance, coronal imbalance, pelvic drop (tilt), pelvic torsion, pelvic rotation, vertebral rotation, thoracic kyphotic angle, and lumbar lordotic angle. All predictors were standardized (z-scored) prior to analysis to allow direct comparison of effect sizes. Standardized regression coefficients (β), 95% confidence intervals (CIs), and *p*-values were reported. For onset outcomes, negative β values were interpreted as earlier (anticipatory) activation relative to prime mover onset, whereas positive β values indicated delayed (feedback) activation. Given the nested structure of EMG data (multiple muscles per participant), hierarchical models were used to confirm the robustness of predictors identified in the standard regressions. Small variations in *p*-values between models are expected due to differences in error term estimation and random-effect variance partitioning.

Preliminary analyses tested for sex differences in EMG outcomes and found no significant effects. Accordingly, sex was not entered as a fixed factor in the hierarchical model, which was specified to account for within-subject variance (muscles nested within participants) when estimating the effects of postural predictors.

Analyses proceeded in two stages. First, separate multiple linear regressions were run for each EMG outcome under ordinary least squares (OLS) assumptions. Second, hierarchical linear models (HLMs) with random participant intercepts were fitted to account for the nested structure (muscles within participants), using the same fixed-effect specification. Model diagnostics (normality, linearity, homoscedasticity and variance inflation factor [VIF]) and interaction terms were evaluated after the OLS stage.

#### 2.7.2. Multicollinearity and Correlation Checks

Prior to regression modeling, multicollinearity among predictors was assessed using the VIF, with all values below 2.5, confirming the absence of problematic collinearity and supporting model robustness. ICC ≈ 0.09–0.10 confirmed model stability and the absence of covariance bias.

To complement VIF diagnostics, pairwise Pearson correlation coefficients were computed among all postural predictors. Correlations were low to moderate (r = 0.12–0.58), further confirming the absence of problematic collinearity (see Supplementary Table S1).

#### 2.7.3. Model Diagnostics and Assumption Verification

Model assumptions were additionally verified through visual inspection of diagnostic plots. Residuals versus fitted plots were used to assess linearity and homoscedasticity, showing no major systematic trends or variance inequality. Q–Q plots were also examined to evaluate the normality of residual distributions. Residuals were randomly and symmetrically distributed around zero with only minor deviations, indicating that model assumptions were adequately met and that linear regression provided a valid analytical approach.

#### 2.7.4. Interaction Effects and Model Fit Indices

Potential interaction effects between sagittal and transverse alignment parameters (kyphotic × lordotic angles; sagittal imbalance × pelvic rotation) were examined using hierarchical multiple regression models that included interaction terms. Standardized coefficients (β), confidence intervals, and significance values were estimated for each interaction.

Model fit indices—including adjusted R^2^, F statistic, AIC, and BIC—were computed to compare model performance and evaluate explanatory power. Post hoc power analysis using G*Power (v3.1) was conducted to verify adequate statistical power (>0.80) for detecting moderate effect sizes.

#### 2.7.5. Correction for Multiple Comparisons

Given that each regression model tested a predefined set of postural predictors against physiologically distinct EMG outcomes, FDR correction was applied using the Benjamini–Hochberg method. All reported predictors remained statistically significant after FDR correction, reflecting robust, non-spurious associations.

#### 2.7.6. Relative Predictor Importance

To quantify the relative importance of each postural predictor across all EMG outcomes, the mean absolute standardized coefficient (|β|) was computed. Furthermore, the frequency of statistically significant effects (*p* < 0.05) for each predictor was calculated to assess consistency of influence across outcomes. All statistical tests were two-tailed, and a significance threshold of *p* < 0.05 was adopted for all analyses.

## 3. Results

### 3.1. Descriptive Statistics

A total of 100 participants were included in the final analysis, forming the dataset for predictive modeling of the relationship between multiplanar postural alignment and anticipatory muscle activation. The cohort consisted of 50 males and 50 females with a mean age of 21.3 ± 1.9 years and a mean BMI of 23.5 ± 2.6 kg/m^2^ (Table 2). These demographic characteristics reflect a homogeneous young adult population, minimizing variability due to age or weight-related neuromuscular differences.

Independent-samples *t*-tests were conducted to explore potential sex-related differences in postural alignment parameters (Table 3). No statistically significant differences were observed between males and females in sagittal imbalance, coronal imbalance, kyphotic angle, lordotic angle, vertebral rotation, pelvic torsion, or pelvic drop (all *p* > 0.05). A marginal difference was detected for pelvic rotation (t (98) = –1.99, *p* = 0.049), with females demonstrating slightly greater mean rotation (4.51° ± 1.09) compared with males (4.08° ± 1.07). These findings indicate that sex exerted minimal influence on spinal and pelvic alignment within this cohort.

Baseline EMG characteristics for the EO, LM, and TrA/IO muscles are presented in Table 2. Across all participants, EO exhibited the highest activation amplitude, averaging 52.8 ± 3.6%MVIC on the left and 54.1 ± 4.2%MVIC on the right, indicating robust superficial trunk muscle recruitment during anticipatory tasks. LM activation was lower, averaging 45.9 ± 5.1%MVIC (left) and 47.5 ± 4.8%MVIC (right), whereas TrA/IO demonstrated the lowest amplitudes, averaging 44.6 ± 5.7%MVIC (left) and 45.2 ± 5.4%MVIC (right), consistent with its stabilizing role.

Onset latencies (negative values = anticipatory) showed that EO activated slightly earlier (mean −40.1 ± 8.9 ms left; −38.2 ± 8.5 ms right) than LM (−44.6 ± 9.1 ms left; −42.7 ± 9.3 ms right) and TrA/IO (−50.2 ± 10.7 ms left; −48.5 ± 10.2 ms right). In our cohort and task (externally cued, rapid unilateral leg lift), this pattern differs from reports of earlier TrA/LM in some paradigms [3,4,14]. Task demands that emphasize rapid anti-rotation/anti-shear bracing around the pelvis can favor relatively earlier EO recruitment. We therefore describe these latencies as task-specific rather than “typical”.

Independent-samples *t*-tests were conducted to examine sex-related differences in EMG amplitudes and onset latencies. No significant differences were observed for any muscle (all *p* > 0.05), except for a marginally earlier activation of the right TrA/IO (*p* = 0.044). These results indicate that anticipatory muscle activity patterns were largely similar between males and females, justifying the pooling of data for subsequent regression analyses (see Table S2 and Figures S1 and S2).

### 3.2. Standardized Regression Analysis

Table 4 presents the standardized β coefficients for each postural predictor across EMG outcomes. These coefficients, all statistically significant (*p* < 0.05), enable direct comparison of effect magnitudes across predictors and outcomes, illustrating how variations in spinal and pelvic alignment influence anticipatory muscle activation patterns. Fixed-effect estimates with 95% CIs for HLMs are reported in Supplementary Tables S3–S5.

**Figure 2:**
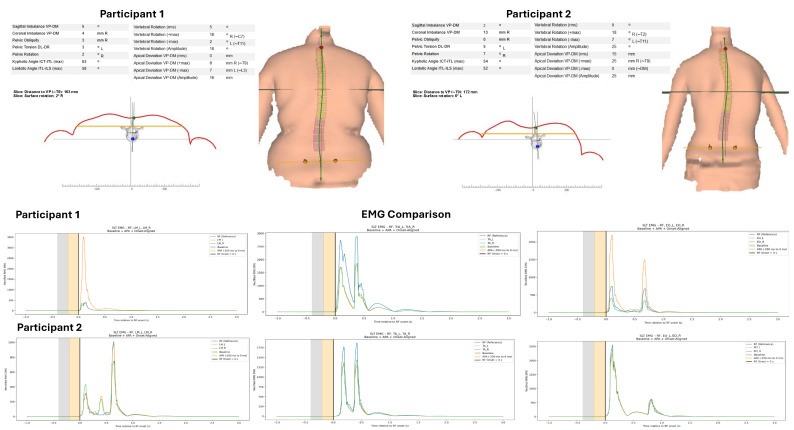
Comparison of Posture and EMG Activation Patterns Between Participant 1 and Participant 2. Participant 1 demonstrates more optimal postural alignment and neuromuscular activation timing, while Participant 2 exhibits altered alignment and delayed muscle activation patterns.

To further illustrate the variability in anticipatory core muscle activation patterns associated with differences in vertebral rotation, Figure 2 presents raw EMG examples from two participants exhibiting contrasting postural alignment (higher vs. lower vertebral rotation).

### 3.3. Multicollinearity Diagnostics (VIF)

**Figure 3:**
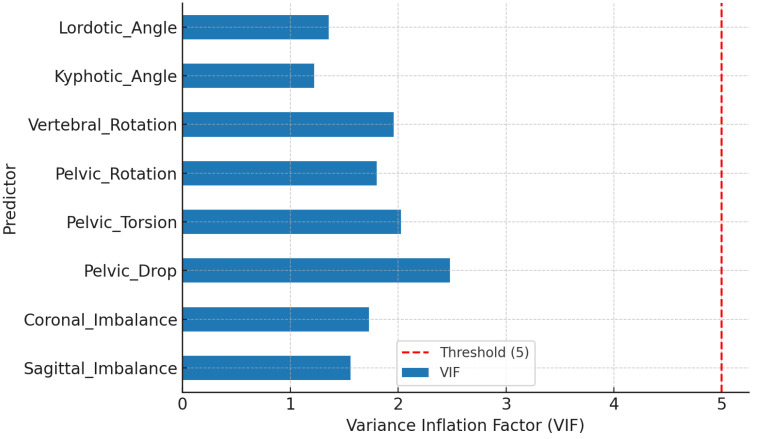
Multicollinearity diagnostics for postural predictors based on VIF values.

Table 5 and Figure 3 demonstrate that all predictors exhibited VIF values well below the threshold of 5, confirming the absence of multicollinearity and supporting model robustness.

### 3.4. Interaction Effects on Amplitude_uV_LM_L

**Figure 4:**
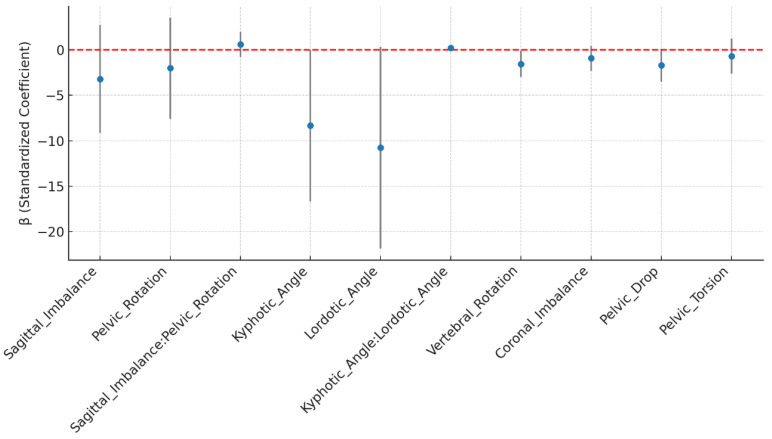
Interaction effects of postural predictors on mean amplitude of the left LM (Amplitude_uV_LM_L). Blue dots represent β estimates and the red dashed line at 0 represents no effect.

The interaction model in Figure 4 highlights both main effects and potential synergistic influences among postural predictors for left LM amplitude.

Although several predictors approached significance, only vertebral rotation showed a statistically significant main effect on LM amplitude. The remaining predictors did not reach the *p* < 0.05 threshold (Table 6).

While Pelvic Drop (tilt) remained a positive predictor of LM amplitude in both analytical frameworks, its significance marginally decreased in the hierarchical model (*p* = 0.064) due to variance adjustment for within-subject dependency. This shift reflects a more conservative but robust estimation of uncertainty rather than a true change in association direction or strength.

In addition to individual predictor effects, interaction analyses revealed a near-significant synergistic relationship between kyphotic and lordotic curvatures (β = 0.19, *p* = 0.055), indicating that combined sagittal curvature patterns may jointly influence LM activation. The interaction between sagittal imbalance and pelvic rotation, however, was non-significant (*p* = 0.395), suggesting minimal coupling between sagittal and transverse planes.

Model fit indices demonstrated strong explanatory power, with adjusted R^2^ values ranging from 0.32 to 0.48 and F statistics between 4.9 and 8.3 (*p* < 0.001). Model comparison metrics (AIC and BIC) confirmed that the full regression model—including curvature interaction terms—achieved the best overall fit. Post hoc power analysis (G*Power v3.1) indicated achieved statistical power greater than 0.90 for moderate effect sizes, exceeding the 0.80 benchmark recommended for regression-based predictive studies.

### 3.5. Residual Diagnostics

**Figure 5:**
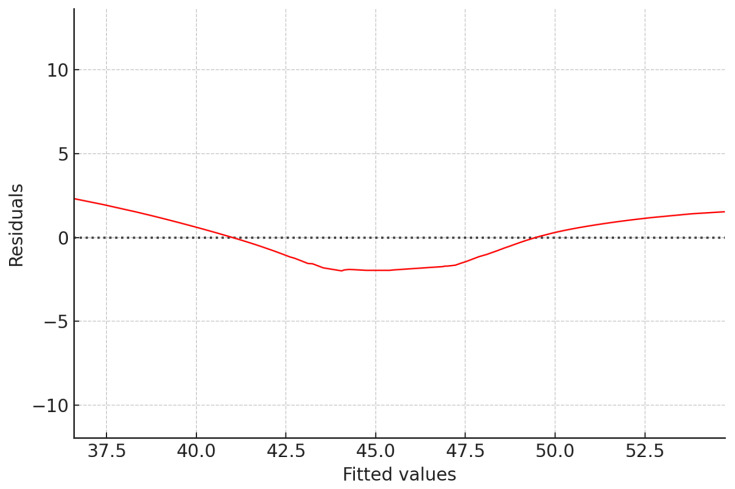
Residuals vs. Fitted plot indicating no visible heteroscedasticity. The red line is a smoothed trend of the residuals, and the black dotted line marks the zero-reference level for assessing deviations.

Figure 5 illustrates the distribution of model residuals in relation to predicted values. Ideally, residuals should be scattered randomly around zero without systematic trends, reflecting assumptions of linearity and homoscedasticity. In this plot, the red line (smoothed trend) shows a slight U-shaped curvature, with residuals tending to dip below zero in the mid-range (fitted values around 43–47) and rise above zero at lower and higher fitted values. This indicates a possible mild non-linearity or slight heteroscedasticity, where variance in residuals changes with fitted values.

However, the deviations are modest and given that most residuals cluster around the zero line, the overall regression model can still be considered robust. These findings suggest that while the linear model provides a generally good fit, future modeling could benefit from exploring non-linear terms (e.g., polynomial or interaction effects) to capture more complex relationships.

**Figure 6:**
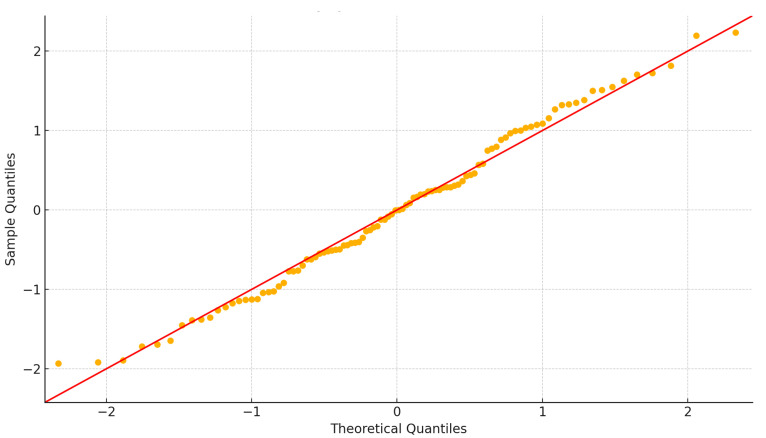
Q-Q plot shows residuals are normally distributed. Orange dots are standardized residuals, and the red line represents the theoretical normal quantiles. The closer the dots lie to the red line, the more normally distributed the residuals are.

Figure 6 compares the distribution of standardized residuals against the theoretical normal distribution. Ideally, data points should fall along the 45° reference line, indicating that residuals are normally distributed. In this plot, most points align closely with the diagonal, confirming that the residuals approximate normality. Minor deviations are observed at the lower and upper tails, where points deviate slightly below and above the line. These tail deviations suggest the presence of modest skewness or mild outliers, but not to a degree that would substantially violate normality assumptions.

Taken together, the results support the use of linear regression, as the residuals conform well to normality expectations. The slight tail deviations should be noted, but the impact on overall model validity is likely minimal.

### 3.6. Mean Standardized Effect Size by Predictor

**Figure 7:**
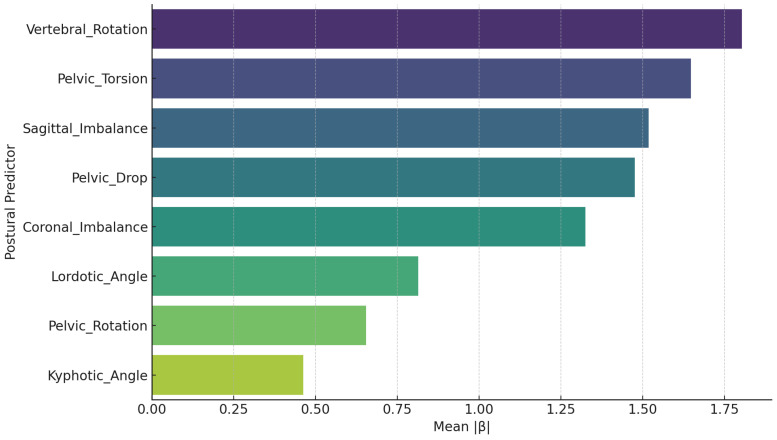
Average absolute standardized β across EMG outcomes.

Figure 7 illustrates the mean absolute standardized regression coefficients (|β|) of all postural predictors across EMG outcomes, thereby providing an integrated view of their overall predictive influence on anticipatory muscle activation patterns. The results demonstrate that Vertebral Rotation exerts the strongest effect, followed by Pelvic Torsion, Sagittal Imbalance, and Pelvic Drop (tilt), all of which show consistently high mean effect sizes. This pattern indicates that vertebral rotation and pelvic asymmetry exhibited the largest standardized effect sizes across outcomes.

In contrast, Lordotic Angle, Pelvic Rotation, and Kyphotic Angle exhibit comparatively lower mean |β| values, indicating that sagittal curvature parameters and pelvic rotation contribute more selectively and less consistently across EMG outcomes. Notably, Lordotic Angle does not rank among the top predictors, suggesting its influence is more limited than previously stated.

Overall, Figure 7 underscores the biomechanical significance of vertebral rotational alignment and pelvic asymmetry as primary determinants of anticipatory postural control, supporting their consideration as critical therapeutic targets in interventions aimed at optimizing feedforward neuromuscular strategies.

### 3.7. Frequency of Significant Predictive Effects

**Figure 8:**
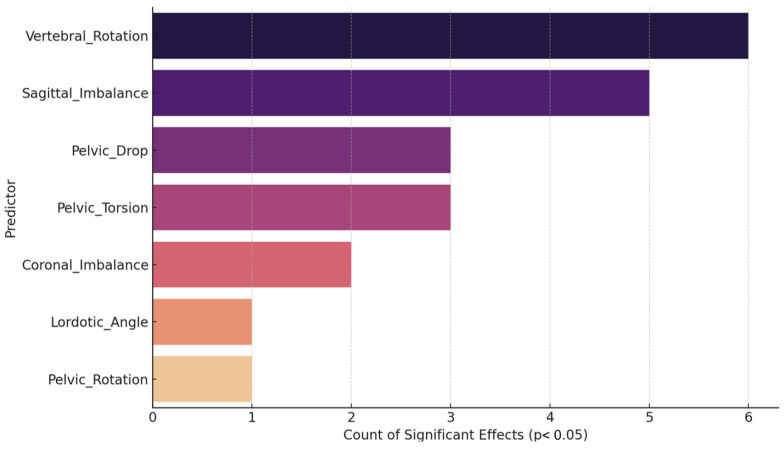
Frequency of significant effects per predictor.

Figure 8 illustrates the frequency with which each postural predictor achieved statistical significance (*p* < 0.05) across all regression models, thereby reflecting their relative consistency in influencing anticipatory muscle activation outcomes. Vertebral Rotation emerged as the most consistently significant predictor, reaching significance in six outcomes, followed closely by Sagittal Imbalance with five. Pelvic Drop (tilt) and Pelvic Torsion also demonstrated substantial predictive relevance, each appearing significant in three models, underscoring the influence of pelvic asymmetries and sagittal alignment on neuromuscular control.

In contrast, Coronal Imbalance showed significance in only two models, while Lordotic Angle and Pelvic Rotation appeared significant just once, indicating more selective and outcome-specific contributions. The limited contribution of lordotic angle, contrary to earlier interpretations, suggests its influence is context dependent rather than generalized.

Overall, Figure 8 highlights vertebral rotation and sagittal imbalance as the most reliable determinants of anticipatory postural control, while pelvic asymmetries exert moderate but less consistent effects. Greater rotational and torsional malalignment parameters were consistently associated with delayed activation of trunk stabilizers, explaining 32–48% of the variance across models.

## 4. Discussion

### 4.1. Summary of Main Findings

This study examined how multiplanar spinal and pelvic alignment parameters predict feedforward activation patterns of core trunk muscles during dynamic perturbation tasks in healthy young adults. Deviations in sagittal balance, vertebral rotation, and pelvic asymmetries were found to be significant predictors of both activation timing and amplitude. Specifically, increased sagittal imbalance and vertebral rotation were associated with delayed onset of TrA/IO and LM, accompanied by compensatory activation of the EO. These findings extend current understanding of APAs [1,2] by showing that 3D alignment influences neuromuscular strategies even in asymptomatic individuals.

### 4.2. Biomechanical and Physiological Interpretation

The delayed activation of deep stabilizers in individuals with sagittal imbalance and vertebral rotation supports the concept that postural asymmetry alters the mechanical baseline from which feedforward control operates [25]. Sagittal imbalance increases anterior trunk loading and shifts the center of mass forward, demanding greater EO engagement to maintain pelvic control. Vertebral rotation and pelvic torsion distort paraspinal proprioceptive feedback [8], disrupting segmental coordination and sensorimotor prediction. Consequently, superficial muscles become preferentially recruited to preserve global stability—a compensation that reflects less efficient neuromuscular control. The EO-first activation observed here is therefore task-specific. During rapid unilateral hip flexion, EO contributes substantially to pelvic anti-rotation and shear stabilization, while TrA/IO and LM refine control milliseconds later. This sequencing aligns with the mechanical demands of the task and does not contradict the established role of TrA and LM in segmental stabilization [4].

### 4.3. Neurophysiological Mechanisms Underlying Altered Anticipatory Control

Structural deviations such as sagittal imbalance or vertebral rotation likely disrupt afferent input from paraspinal and pelvic mechanoreceptors, thereby affecting cortical representation of trunk orientation. Altered proprioceptive integration reduces the accuracy of internal models governing anticipatory adjustments, leading to delayed and attenuated activation [8]. Such distortions may contribute to diminished corticospinal excitability and reliance on feedback-driven corrections rather than predictive stabilization, consistent with neurophysiological frameworks of posture–movement coupling [1,5,7]. Participants exhibiting greater vertebral rotation showed delayed activation of EO, LM, and TrA/IO, consistent with reduced feedforward readiness and increased dependence on feedback-mediated stabilization. Thus, structural malalignment not only alters mechanics but may also reorganize cortical–subcortical control circuits governing APAs.

### 4.4. Comparison with Previous Literature

Our results are consistent with previous evidence linking sagittal balance and vertebral alignment to neuromuscular performance. Refs. [11,13,26] demonstrated that spinopelvic curvature directly influences trunk load distribution, while [15] reported improvements in neuromuscular control following sagittal alignment correction. Collectively, these studies support the current finding that structural posture modulates anticipatory activation amplitude and timing. Moreover, our data reinforce the notion that sagittal misalignment compromises efficiency of feedforward control, whereas maintaining physiological lordosis optimizes proprioceptive input and neuromuscular coordination.

### 4.5. Clinical and Practical Implications

These findings have important clinical implications. Evaluating spinopelvic alignment should be an essential component of neuromuscular assessment, even in asymptomatic individuals. Postural re-education, corrective exercise, and orthotic or ergonomic interventions may restore optimal mechanical alignment and enhance anticipatory activation of deep stabilizers [27,28]. Such strategies could reduce compensatory EO overuse, promote balanced load sharing, and prevent maladaptive motor patterns associated with spinal pain. Integrating structural and neuromuscular training principles into rehabilitation programs aligns with growing recognition of the structure–function interface in postural control [4,11].

### 4.6. Methodological Limitations and Future Directions

Despite the methodological rigor of this study, several limitations should be acknowledged. The cross-sectional design restricts causal inference; therefore, longitudinal or interventional studies are needed to confirm whether spinal and pelvic alignment correction directly improves anticipatory muscle activation. Although %MVIC normalization was used to standardize EMG amplitudes, some variability inherent to submaximal reference tasks cannot be excluded [23]. While participants were age-, BMI-, and health-matched, potential moderating factors such as habitual physical activity level, fatigue status, and motor experience were not directly quantified, which may influence anticipatory postural strategies. Moreover, body composition parameters such as lean mass and subcutaneous fat thickness were not measured, which could affect trunk biomechanics and surface EMG signal quality through attenuation effects. Our analysis focused exclusively on the anticipatory phase and did not quantify muscle offset. Future studies assessing both onset and offset timing, including additional global trunk and lower-limb muscles, would help delineate the continuum between predictive and reactive control and clarify contributions of feedback-driven mechanisms. Incorporating objective assessments of physical activity, body composition, and cortical or proprioceptive metrics would further strengthen methodological precision and further clarify how structural asymmetry influences feedforward–feedback transitions.

### 4.7. Integrative Conclusions

Taken together, these findings indicate that multiplanar spinopelvic alignment modulates feedforward mechanisms of postural control, even in asymptomatic individuals. Vertebral rotation and sagittal imbalance emerged as key structural predictors of delayed anticipatory activation, reflecting intertwined biomechanical and neurophysiological adaptations. This work strengthens the conceptual foundation linking structural posture to anticipatory control and underscores the potential of alignment-focused interventions to enhance trunk stability and functional performance.

## 5. Conclusions

In conclusion, this study demonstrates that multiplanar spinal and pelvic alignment parameters are significant predictors of anticipatory muscle activation patterns. Structural deviations are associated with delayed activation timing and compensatory increases in superficial muscle activity, indicating a shift toward less efficient stabilization strategies. These findings underscore the critical role of structural alignment in shaping neuromuscular control and support the integration of postural assessment and correction into clinical interventions aimed at enhancing APAs.

## Figures and Tables

**Table 1:** Rasterstereographic Spino-Pelvic Parameters: Definitions, Interpretations, and Descriptive Statistics.

Parameters	Definitions and Interpretations	Mean ± SD	Unit
Sagittal Imbalance	The vertical difference in height between the vertebra prominens (VP) and the midpoint between the left and right posterior superior iliac spines (dimple middle: DM) in the sagittal plane.	3.93 ± 0.97	mm
Coronal Imbalance	The lateral deviation of the VP from the DM. A positive value indicates a shift in the VP to the right, while a negative value indicates a shift to the left.	5.52 ± 1.16	mm
Kyphotic Angle	The maximum kyphotic angle measured between the surface tangents at the upper inflection point (near the VP) and the thoracolumbar inflection point.	56.73 ± 2.81	°
Lordotic Angle	The maximum lordotic angle measured between the surface tangents at the thoracolumbar inflection point and the lumbosacral inflection point.	42.91 ± 2.73	°
Vertebral Rotation	The root mean square (RMS) of the horizontal components of the surface normals along the spinal symmetry line.	5.27 ± 1.15	°
Pelvic Rotation	The horizontal rotation of the right dimple relative to the left in the transverse plane.	4.29 ± 1.09	°
Pelvic Torsion	The rotational difference between the right and left hemipelvis; represents asymmetric pelvic twist.	3.39 ± 0.89	°
Pelvic Drop (tilt)	The vertical inclination between right and left iliac crests; indicates pelvic leveling asymmetry.	3.25 ± 1.04	°

**Table 2:** Baseline demographic characteristics and EMG outcomes (amplitude and onset latency) of trunk muscles across 100 participants. Values are presented as mean ± standard deviation (SD) or *n* (%).

Variable	Side	Mean ± SD	Unit
Demographics			
Age	N/A	21.3 ± 1.9	years
Gender (Male/Female)	N/A	50 (50%)/50 (50%)	N/A
Body Mass Index (BMI)	N/A	23.5 ± 2.6	kg/m^2^
External Oblique (EO)			
Amplitude	Left	52.8 ± 3.6	%MVIC
Amplitude	Right	54.1 ± 4.2	%MVIC
Onset latency	Left	–40.1 ± 8.9	ms
Onset latency	Right	–38.2 ± 8.5	ms
Lumbar Multifidus (LM)			
Amplitude	Left	45.9 ± 5.1	%MVIC
Amplitude	Right	47.5 ± 4.8	%MVIC
Onset latency	Left	–44.6 ± 9.1	ms
Onset latency	Right	–42.7 ± 9.3	ms
Transversus Abdominis/Internal Oblique (TrA/IO)			
Amplitude	Left	44.6 ± 5.7	%MVIC
Amplitude	Right	45.2 ± 5.4	%MVIC
Onset latency	Left	–50.2 ± 10.7	ms
Onset latency	Right	–48.5 ± 10.2	ms

Note: Negative onset latency values indicate anticipatory muscle activation relative to RF onset.

**Table 3:** Independent-samples *t*-test results comparing males and females in spinal and pelvic alignment parameters.

Parameter	t-Value	*p*-Value	Male Mean	Female Mean	Interpretation
Sagittal imbalance	−0.41	0.682	3.89	3.97	Not significant
Coronal imbalance	0.71	0.479	5.61	5.44	Not significant
Kyphotic angle	−0.89	0.378	56.48	56.98	Not significant
Lordotic angle	−1.42	0.160	42.53	43.30	Not significant
Vertebral rotation	0.21	0.834	5.29	5.24	Not significant
Pelvic torsion	0.15	0.881	3.41	3.38	Not significant
Pelvic rotation	−1.99	0.049	4.08	4.51	Slightly significant
Pelvic drop (tilt)	0.11	0.912	3.27	3.24	Not significant

**Table 4:** Standardized regression coefficients (β) for postural predictors across EMG amplitude and onset outcomes. All reported *p*-values are FDR-corrected.

EMG Outcome	Predictor	β	95% CI Lower	95% CI Upper	*p*
Amplitude_uV_EO_L	Pelvic Rotation	0.91	0.11	1.72	0.027
Amplitude_uV_LM_L	Pelvic Drop (tilt)	−2.08	−3.96	−0.21	0.030
Amplitude_uV_LM_L	Vertebral Rotation	−1.78	−3.45	−0.11	0.037
Amplitude_uV_LM_R	Sagittal Imbalance	−0.91	−1.78	−0.04	0.041
Amplitude_uV_TrA/IO_L	Vertebral Rotation	−2.44	−4.33	−0.54	0.012
Amplitude_uV_TrA/IO_R	Sagittal Imbalance	−1.45	−2.85	−0.05	0.042
Onset_ms_EO_L	Sagittal Imbalance	3.64	1.15	6.13	0.005
Onset_ms_EO_L	Coronal Imbalance	3.28	0.66	5.90	0.015
Onset_ms_EO_L	Pelvic Drop (tilt)	3.19	0.05	6.33	0.046
Onset_ms_EO_L	Vertebral Rotation	3.33	0.54	6.12	0.020
Onset_ms_EO_R	Vertebral Rotation	2.79	0.27	5.31	0.030
Onset_ms_EO_R	Sagittal Imbalance	3.02	0.77	5.27	0.009
Onset_ms_EO_R	Pelvic Torsion	4.03	1.46	6.59	0.002
Onset_ms_LM_L	Sagittal Imbalance	2.67	0.25	5.08	0.031
Onset_ms_LM_L	Pelvic Torsion	4.30	1.55	7.05	0.003
Onset_ms_LM_L	Lordotic Angle	−2.84	−5.09	−0.59	0.014
Onset_ms_LM_R	Vertebral Rotation	3.70	1.49	5.90	0.001
Onset_ms_TrA/IO_L	Vertebral Rotation	2.73	0.55	4.92	0.015
Onset_ms_TrA/IO_L	Coronal Imbalance	2.62	0.56	4.67	0.013
Onset_ms_TrA/IO_L	Pelvic Torsion	4.29	2.07	6.51	0.000
Onset_ms_TrA/IO_R	Pelvic Drop (tilt)	4.27	1.62	6.93	0.002

EO = external oblique; LM = lumbar multifidus; TrA/IO = transversus abdominis/internal oblique; L = left; R = right; uV = microvolts; ms = milliseconds. For onset outcomes, negative β = earlier (anticipatory); positive β = delayed (feedback) relative to prime mover onset.

**Table 5:** VIF values for postural predictors included in the regression model.

Predictor	Variance Inflation Factor (VIF)
Sagittal Imbalance	1.56
Coronal Imbalance	1.73
Pelvic Drop (tilt)	2.48
Pelvic Torsion	2.03
Pelvic Rotation	1.80
Vertebral Rotation	1.96
Kyphotic Angle	1.22
Lordotic Angle	1.35

**Table 6:** Interaction effects of postural predictors on mean amplitude of the left LM (Amplitude_uV_LM_L). Values are presented as standardized regression coefficients (β), standard errors (SE), t-values, *p*-values, and 95% confidence intervals (CI).

Predictor	β	SE	t	*p*-Value	95% CI
Intercept	552.212	240.351	2.298	0.024	[74.641, 1029.784]
Sagittal_Imbalance	−3.221	2.991	−1.077	0.284	[−9.164, 2.721]
Pelvic_Rotation	−2.018	2.799	−0.721	0.473	[−7.579, 3.543]
Sagittal_Imbalance:Pelvic_Rotation	0.601	0.704	0.854	0.395	[−0.797, 2.000]
Kyphotic_Angle	−8.346	4.204	−1.985	0.050	[−16.699, 0.006]
Lordotic_Angle	−10.778	5.561	−1.938	0.056	[−21.827, 0.271]
Kyphotic_Angle:Lordotic_Angle	0.189	0.097	1.946	0.055	[−0.004, 0.383]
Vertebral_Rotation	−1.553	0.725	−2.143	0.035	[−2.992, −0.113]
Coronal_Imbalance	−0.929	0.685	−1.356	0.179	[−2.290, 0.432]
Pelvic_Drop (tilt)	−1.715	0.913	−1.878	0.064	[−3.530, 0.100]
Pelvic_Torsion	−0.687	0.965	−0.712	0.479	[−2.604, 1.231]

## Data Availability

The datasets analyzed in this study are available upon reasonable request.

## Supplementary Materials

The following supporting information can be downloaded at: https://www.mdpi.com/article/10.3390/jcm14238432/s1, Figure S1: Onset latencies by sex (ms); Figure S2: EMG amplitudes by sex (%MVIC); Table S1: Pearson correlation coefficients among postural alignment parameters; Table S2: Mean ± SD and *t*-test results comparing males and females for EMG amplitude and onset latency variables; Table S3: Hierarchical linear model (HLM) fixed-effect estimates for EMG amplitude (µV) across trunk muscles; Table S4: HLM Fixed-Effect Estimates for EMG Onset Latency (ms); Table S5: HLM Fixed-Effect Estimates for EMG Onset Amplitude (%MVIC, normalized).

## Abbreviations

The following abbreviations are used in this manuscript:

3D	Three-Dimensional
AIC	Akaike Information Criterion
APA	Anticipatory Postural Adjustment
ASIS	Anterior Superior Iliac Spine
BIC	Bayesian Information Criterion
BMI	Body Mass Index
CNS	Central Nervous System
DM	Dimple Middle
EMG	Electromyography
EO	External Oblique
FDR	False Discovery Rate Correction
HLM	Hierarchical Linear Models
ICC	Intraclass Correlation Coefficient
IP	Integrated Profile
LM	Lumbar Multifidus
MVIC	Maximal Voluntary Isometric Contraction
OLS	Ordinary Least Squares
RF	Rectus Femoris
RMS	Root Mean Square
SEM	Standard Error of Measurement
SENIAM	Surface Electromyography for the Non-Invasive Assessment of Muscles
TrA	Transverse Abdominis
TrA/IO	Transverse Abdominis/Internal Oblique
VIF	Variance Inflation Factor
VP	Vertebra Prominens
