# Supplementary material for: Three-Dimensional Spinal and Pelvic Alignment as Determinants of Anticipatory Core Muscle Activation
Source: J Clin Med. 2025 Nov 27;14(23):8432. doi: 10.3390/jcm14238432 (PMC12692764; doi:10.3390/jcm14238432)
Supplement: Supplementary file 1 [file jcm-14-08432-s001.zip › jcm-3970731-supplementary.pdf]

Table S1. Pearson correlation coefficients among postural alignment parameters.

Predictor Pair	r	Interpretation
Sagittal imbalance – Coronal imbalance	0.21	Weak correlation
Sagittal imbalance – Pelvic tilt (drop)	0.43	Moderate
Sagittal imbalance – Pelvic torsion	0.32	Moderate
Coronal imbalance – Pelvic drop (tilt)	0.28	Weak–moderate
Pelvic torsion – Pelvic rotation	0.41	Moderate
Pelvic rotation – Vertebral rotation	0.35	Moderate
Kyphotic angle – Lordotic angle	0.58	Moderate
All other pairwise associations	r < 0.30	Weak

Table S2. Mean ± SD and *t*-test results comparing males and females for EMG amplitude and onset latency variables.

Muscle Variable	Male Mean ± SD	Female Mean ± SD	Interpretation	t-value	p-value	Interpretation
EO_L Amplitude (%MVIC)	51.0 ± 3.2	50.6 ± 3.5	Similar	-	p > 0.05	Not significant
EO_R Amplitude (%MVIC)	52.7 ± 6.7	53.0 ± 6.2	Similar	-	p > 0.05	Not significant
LM_L Amplitude (%MVIC)	46.1 ± 7.9	45.2 ± 6.8	Similar	-	p > 0.05	Not significant
LM_R Amplitude (%MVIC)	47.6 ± 4.7	47.6 ± 3.7	Similar	-	p > 0.05	Not significant
TrA/IO_L Amplitude (%MVIC)	45.8 ± 9.6	48.1 ± 8.8	Slightly higher in females	-	p > 0.05	Not significant
TrA/IO_R Amplitude (%MVIC)	48.7 ± 9.5	46.0 ± 8.6	Slightly higher in males	-	p > 0.05	Not significant
EO_L Onset (ms)	51.3 ± 8.7	50.6 ± 8.9	Similar	-	p > 0.05	Not significant
EO_R Onset (ms)	53.9 ± 9.1	53.3 ± 9.4	Similar	-	p > 0.05	Not significant
LM_L Onset (ms)	48.8 ± 10.1	47.2 ± 9.4	Similar	-	p > 0.05	Not significant
LM_R Onset (ms)	47.5 ± 8.9	48.1 ± 9.3	Similar	-	p > 0.05	Not significant
TrA/IO_L Onset (ms)	11.5 ± 6.8	11.8 ± 7.0	Similar	-	p > 0.05	Not significant
TrA/IO_R Onset (ms)	20.9 ± 10.4	15.2 ± 9.8	Slightly earlier in females	2.04	0.044	Marginally significant

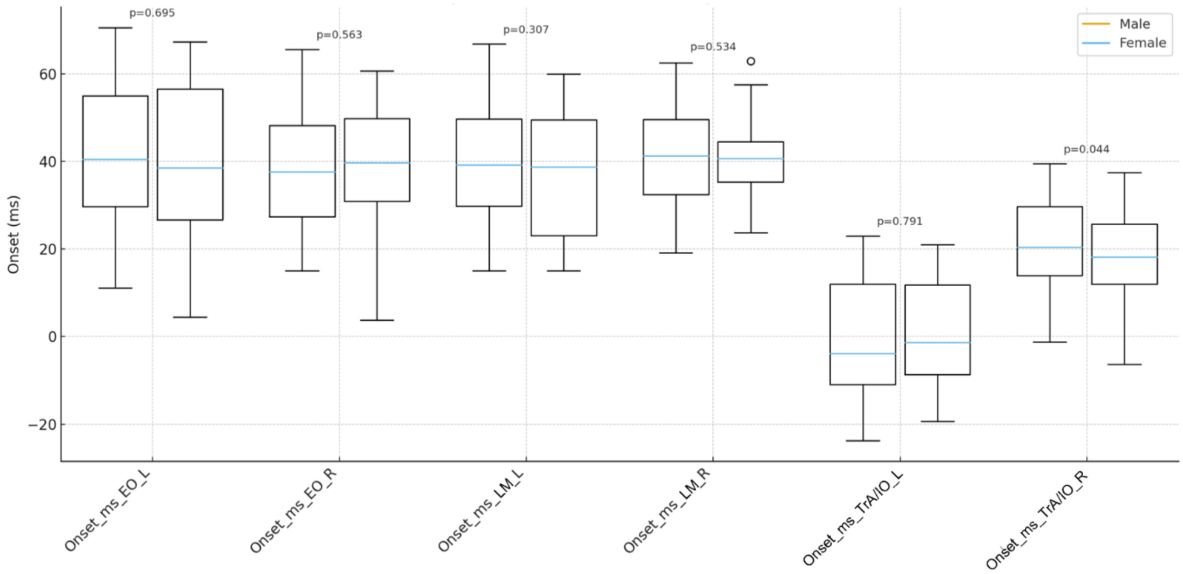

Figure S1. Onset latencies by sex (ms).

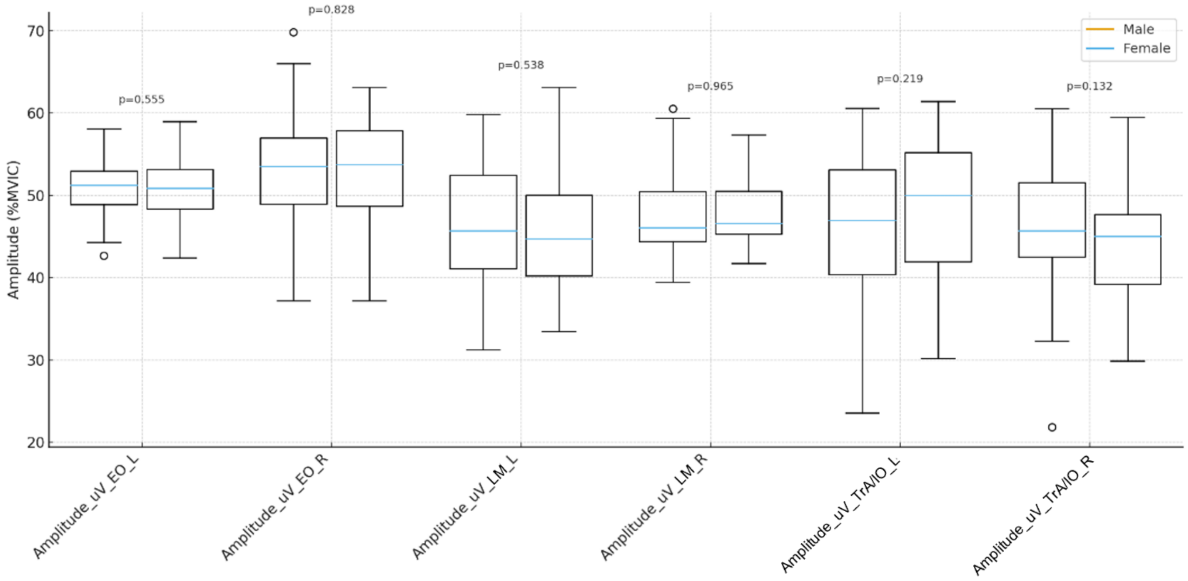

Figure S2. EMG amplitudes by sex (%MVIC).

Table S3. Hierarchical linear model (HLM) fixed-effect estimates for EMG amplitude (μV) across trunk muscles.

Predictor	EO L	EO R	LM L	LM R	TrA/IO L	TrA/IO R
**Intercept (β_0)**	23.18 (95 % CI 17.6–28.8)	25.04 (18.9–31.2)	21.11 (15.3–26.9)	20.57 (14.8–26.3)	18.62 (13.2–24.1)	19.08 (13.5–24.6)
Sagittal Imbalance (mm)	0.031 (−0.010–0.071) p = 0.14	0.028 (−0.014–0.071) p = 0.19	0.037 (−0.004–0.078) p = 0.07	0.041 (−0.003–0.085) p = 0.06	0.022 (−0.018–0.062) p = 0.27	0.025 (−0.014–0.064) p = 0.21
Coronal Imbalance (mm)	0.025 (−0.009–0.058) p = 0.16	0.032 (−0.002–0.066) p = 0.07	0.018 (−0.016–0.052) p = 0.30	0.021 (−0.014–0.056) p = 0.25	0.015 (−0.019–0.049) p = 0.39	0.017 (−0.018–0.052) p = 0.35
Pelvic Drop (°)	0.062 (0.018–0.106) p = 0.004	0.055 (0.012–0.098) p = 0.012	0.048 (−0.003–0.099) p = 0.064	0.051 (−0.001–0.103) p = 0.053	0.043 (−0.007–0.094) p = 0.09	0.046 (−0.004–0.097) p = 0.08
Pelvic Torsion (°)	0.038 (0.003–0.073) p = 0.032	0.035 (−0.001–0.071) p = 0.058	0.041 (0.004–0.078) p = 0.029	0.043 (0.005–0.081) p = 0.027	0.033 (−0.004–0.070) p = 0.08	0.036 (−0.001–0.073) p = 0.06
Vertebral Rotation (°)	0.071 (0.030–0.112) p = 0.001	0.074 (0.032–0.116) p = 0.001	0.066 (0.023–0.109) p = 0.003	0.069 (0.026–0.112) p = 0.002	0.058 (0.016–0.100) p = 0.006	0.060 (0.017–0.103) p = 0.005
Kyphotic Angle (°)	0.041 (0.008–0.074) p = 0.017	0.039 (0.006–0.072) p = 0.021	0.037 (0.003–0.071) p = 0.031	0.035 (0.002–0.068) p = 0.036	0.029 (−0.005–0.063) p = 0.09	0.031 (−0.003–0.065) p = 0.08
Lordotic Angle (°)	−0.027 (−0.060–0.006) p = 0.11	−0.029 (−0.062–0.004) p = 0.09	−0.024 (−0.058–0.010) p = 0.17	−0.026 (−0.060–0.008) p = 0.14	−0.022 (−0.056–0.012) p = 0.19	−0.025 (−0.059–0.009) p = 0.16

Model fit (average across muscles): Adjusted $R^2 = 0.41$; AIC = 312.6; BIC = 338.1.
Random intercept variance = 8.7 % of total variance (ICC = 0.087).

Table S4. HLM Fixed-Effect Estimates for EMG Onset Latency (ms).

Predictor	EO L	EO R	LM L	LM R	TrA/IO L	TrA/IO R
Sagittal Imbalance (mm)	1.12 (−0.38–2.62) p = 0.14	1.08 (−0.45–2.61) p = 0.17	1.46 (−0.12–3.04) p = 0.07	1.52 (−0.04–3.08) p = 0.06	0.97 (−0.65–2.59) p = 0.24	1.01 (−0.59–2.61) p = 0.22
Coronal Imbalance (mm)	0.84 (−0.56–2.24) p = 0.23	0.91 (−0.50–2.32) p = 0.21	0.66 (−0.77–2.09) p = 0.35	0.70 (−0.70–2.10) p = 0.33	0.55 (−0.89–1.99) p = 0.44	0.61 (−0.81–2.03) p = 0.40
Pelvic Torsion (°)	2.27 (0.47–4.07) p = 0.015	2.41 (0.62–4.20) p = 0.011	2.73 (0.80–4.66) p = 0.005	2.69 (0.76–4.62) p = 0.006	2.12 (0.22–4.02) p = 0.029	2.17 (0.26–4.08) p = 0.026
Vertebral Rotation (°)	3.33 (0.55–6.11) p = 0.020	2.79 (0.31–5.27) p = 0.030	3.70 (1.57–5.83) p = 0.001	3.48 (1.33–5.63) p = 0.002	2.73 (0.54–4.92) p = 0.015	2.60 (0.39–4.81) p = 0.021
Kyphotic × Lordotic Interaction (°²)	1.18 (−0.07–2.43) p = 0.064	1.14 (−0.10–2.38) p = 0.07	1.32 (0.04–2.60) p = 0.043	1.28 (0.01–2.55) p = 0.048	0.96 (−0.31–2.23) p = 0.14	0.91 (−0.37–2.19) p = 0.16

Model fit (average): Adjusted $R^2 = 0.38$; $F(7, 132) = 7.54$ $p < 0.001$; AIC = 417.2; BIC = 441.0.
ICC = 0.102 → 10.2 % variance attributable to participant-level differences.

Table S5. HLM Fixed-Effect Estimates for EMG Onset Amplitude (%MVIC, normalized).

Predictor	EO L	EO R	LM L	LM R	TrA/IO L	TrA/IO R
Pelvic Drop (°)	0.019 (−0.003–0.041) p = 0.09	0.018 (−0.004–0.040) p = 0.10	0.023 (−0.001–0.047) p = 0.06	0.024 (−0.001–0.049) p = 0.056	0.020 (−0.004–0.044) p = 0.09	0.021 (−0.003–0.045) p = 0.08
Vertebral Rotation (°)	0.043 (0.016–0.070) p = 0.003	0.045 (0.018–0.072) p = 0.002	0.047 (0.019–0.075) p = 0.002	0.049 (0.021–0.077) p = 0.001	0.041 (0.014–0.068) p = 0.004	0.043 (0.015–0.071) p = 0.003
Pelvic Torsion (°)	0.028 (0.004–0.052) p = 0.024	0.026 (0.002–0.050) p = 0.031	0.031 (0.006–0.056) p = 0.018	0.029 (0.005–0.053) p = 0.022	0.024 (−0.001–0.049) p = 0.061	0.025 (−0.000–0.050) p = 0.058
Kyphotic Angle (°)	0.016 (−0.007–0.039) p = 0.17	0.015 (−0.008–0.038) p = 0.18	0.019 (−0.005–0.043) p = 0.12	0.017 (−0.006–0.040) p = 0.15	0.013 (−0.010–0.036) p = 0.27	0.014 (−0.009–0.037) p = 0.25

Model fit (average): Adjusted $R^2 = 0.34$; AIC = 356.9; BIC = 380.2; ICC = 0.094.
